# CENTENARIAN PHYSICAL FUNCTION EVOLUTION AND COVID-19 IMPACT: A STUDY IN JAPAN

**DOI:** 10.1093/geroni/igad104.3215

**Published:** 2023-12-21

**Authors:** Xinyu Zhang, Yasuyuki Gondo

**Affiliations:** Osaka University, Suita, Osaka, Japan; Osaka University, Suita, Osaka, Japan

## Abstract

Studies show that centenarians’ physical function and ADL levels improved in the recent cohort. However, it is unclear whether this positive trend has altered due to the COVID-19 pandemic. Recent studies indicated that physical function decreased even in younger older adults because of the limited daily activity during the pandemic period. Is this true for centenarians? This study has two aims: firstly, to investigate whether the physical function of Japanese centenarians has improved over the years; secondly, to examine the impact of COVID-19. We used the data collected from 2014 to the present day in Kyotango City. The City Hall has been collecting data including the functional status of all centenarians who turned 100 years old every year. In this study, we divided nine years into three periods: 2014-2016 (P1), 2017-2019 (P2), and 2020-2022 (P3). For each period the number of participants was 122, 116, and 153. The results revealed that the prevalence of bedridden centenarians showed an upward trend over the years. Concurrently, the proportion of centenarians capable of unrestricted mobility exhibited an upward trajectory from P1 to P2, followed by a subsequent decrease from P2 to P3. In conclusion, if there was no COVID-19 pandemic, the functional level of Japanese centenarians could be improved over the years. At the same time, the gradually increasing trend of bedridden centenarians might indicate intensive medical and institutional care enables centenarians with fragile conditions to survive.

